# Supplementary material for: Synthetic Neuraminidase Vaccine Induces Cross-Species and Multi-Subtype Protection
Source: Vaccines (Basel). 2025 Mar 28;13(4):364. doi: 10.3390/vaccines13040364 (PMC12031341; doi:10.3390/vaccines13040364)
Supplement: Supplementary file 1 [file vaccines-13-00364-s001.zip › vaccines-3505029-supplementary.pdf]

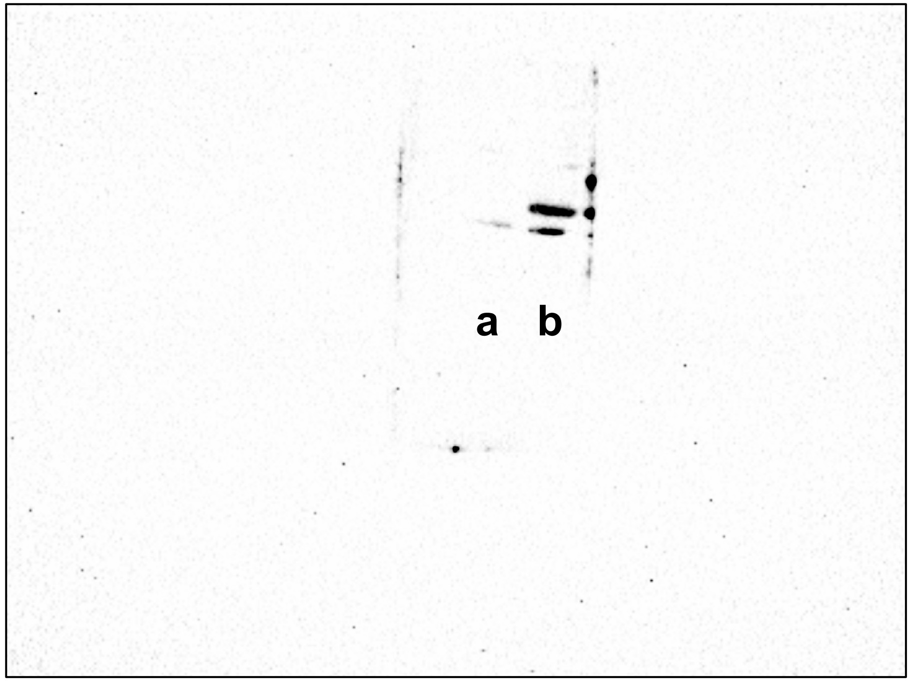

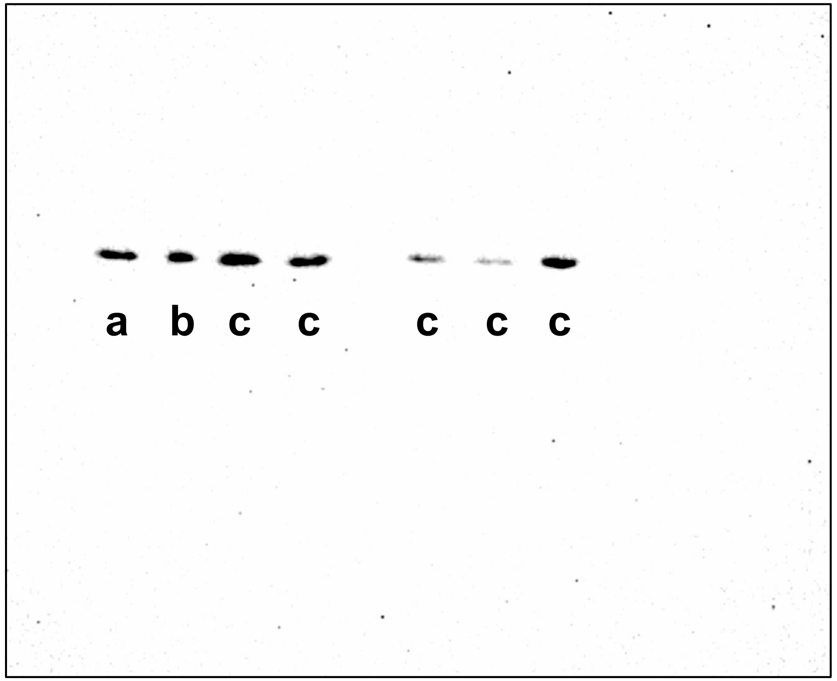

Supplementary Figure 1. Full Western blot images from which Figure 1C were cropped. Left image is blot detecting NA expression using anti-A/New Jersey/8/1976 antiserum as a primary antibody. Right image is blot detecting GAPDH expression using monoclonal anti-GAPDH conjugated with HRP. (a, uninfected; b, N1CC infected; c, unrelated GAPDH samples).
